# Supplementary material for: Evolutionary trajectories of immune escape across cancers
Source: bioRxiv. 2025 Jan 18:2025.01.17.632799. Preprint. [Version 1] doi: 10.1101/2025.01.17.632799 (PMC11761017; doi:10.1101/2025.01.17.632799)
Supplement: Supplement 1 — Figure S1. Overview of antigen presentation machinery mutations in PCAWG. (A) Total number of WGS samples included in the PCAWG dataset. (B) HLA mutation calling by Polysolver and HLA LOH calling by LOHHLA in PCAWG-TCGA samples. (C) dN/dS ratios for antigen presentation machinery mutations in each PCAWG cancer type. Figure S2. Temporal order of antigen presentation machinery mutations and key events in cancers. (A) Distribution of timing of antigen presentation machinery point mutation across cancer types. The plots show the mean timing of 250 timing samples for all APM mutations in each sample. (B) Distribution of timing of HLA LOH, and WGD across cancer types. Figure S3. Determining genetic pathways with immunomodulatory effects. (A) Summary of CRISPR screen studies reporting the enrichments of regulators for the response of cancer cells to NK-cell killing. (B) Summary of CRISPR screen studies reporting the enrichments of regulators for the response of cancer cells to macrophage killing. (C) Summary of CRISPR screen studies reporting the enrichments of regulators for the response of cancer cells to γδ T-cell killing. Figure S4. Prevalence of mutations in genetic pathways with immunomodulatory effects. (A) Prevalence of mutations in the key pathway for regulators of MHC-I regulation. (B) Prevalence of mutations in the key pathway for regulators for the response of cancer cells to NK-cell-mediated killing. (C) Prevalence of mutations in the key pathway for regulators for the response of cancer cells to macrophage-mediated killing. (D) Prevalence of mutations in the key pathway for regulators for the response of cancer cells to γδT-cell-mediated killing. Top panels show the prevalence of mutations in the key pathways for T cell killing to tumor cells in all PCAWG cancer samples. Bottom panels show the prevalence of mutations in the key pathways for T cell killing to tumor cells in different cancer types. Figure S5. Positive selection of mutations in genetic pathways [file media-1.zip › SFigures/SFigure3.pdf]

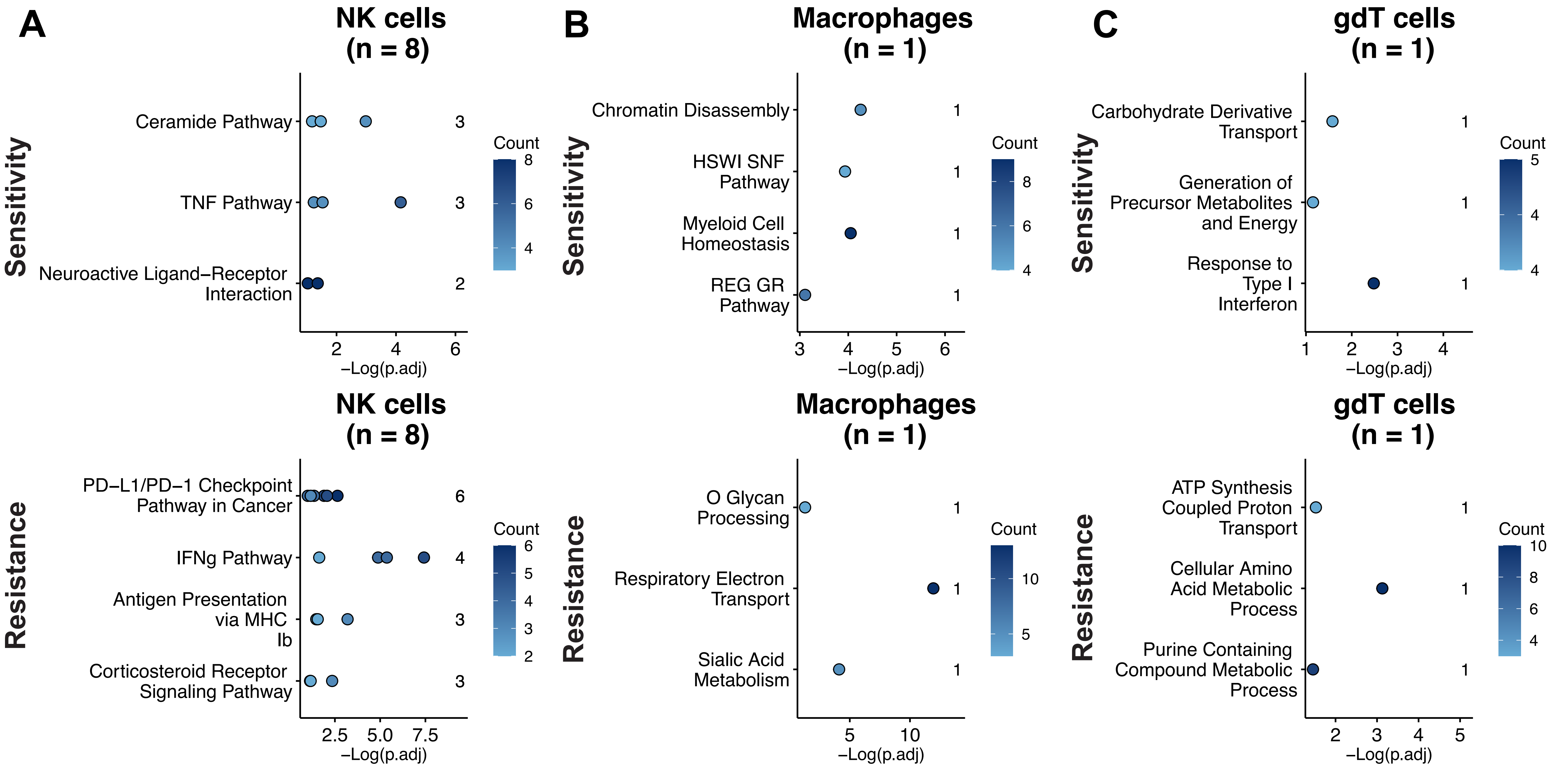

A
NK cells (n = 8)
Sensitivity
Ceramide Pathway
TNF Pathway
Neuroactive Ligand−Receptor Interaction
3
3
2
Count
8
6
4
2
4
6
−Log(p.adj)
B
Macrophages (n = 1)
Sensitivity
Chromatin Disassembly
HSWI SNF Pathway
Myeloid Cell Homeostasis
REG GR Pathway
1
1
1
1
Count
8
6
4
3
4
5
6
−Log(p.adj)
C
gdT cells (n = 1)
Sensitivity
Carbohydrate Derivative Transport
Generation of Precursor Metabolites and Energy
Response to Type I Interferon
1
1
1
Count
5
4
4
1
2
3
4
−Log(p.adj)
NK cells (n = 8)
Resistance
PD−L1/PD−1 Checkpoint Pathway in Cancer
IFNg Pathway
Antigen Presentation via MHC Ib
Corticosteroid Receptor Signaling Pathway
6
4
3
3
Count
6
5
4
3
2
2.5
5.0
7.5
−Log(p.adj)
Macrophages (n = 1)
Resistance
O Glycan Processing
Respiratory Electron Transport
Sialic Acid Metabolism
1
1
1
Count
10
5
5
10
−Log(p.adj)
gdT cells (n = 1)
Resistance
ATP Synthesis Coupled Proton Transport
Cellular Amino Acid Metabolic Process
Purine Containing Compound Metabolic Process
1
1
1
Count
10
8
6
4
2
3
4
5
−Log(p.adj)
