# Supplementary material for: Evolutionary trajectories of immune escape across cancers
Source: bioRxiv. 2025 Jan 18:2025.01.17.632799. Preprint. [Version 1] doi: 10.1101/2025.01.17.632799 (PMC11761017; doi:10.1101/2025.01.17.632799)
Supplement: Supplement 1 — Figure S1. Overview of antigen presentation machinery mutations in PCAWG. (A) Total number of WGS samples included in the PCAWG dataset. (B) HLA mutation calling by Polysolver and HLA LOH calling by LOHHLA in PCAWG-TCGA samples. (C) dN/dS ratios for antigen presentation machinery mutations in each PCAWG cancer type. Figure S2. Temporal order of antigen presentation machinery mutations and key events in cancers. (A) Distribution of timing of antigen presentation machinery point mutation across cancer types. The plots show the mean timing of 250 timing samples for all APM mutations in each sample. (B) Distribution of timing of HLA LOH, and WGD across cancer types. Figure S3. Determining genetic pathways with immunomodulatory effects. (A) Summary of CRISPR screen studies reporting the enrichments of regulators for the response of cancer cells to NK-cell killing. (B) Summary of CRISPR screen studies reporting the enrichments of regulators for the response of cancer cells to macrophage killing. (C) Summary of CRISPR screen studies reporting the enrichments of regulators for the response of cancer cells to γδ T-cell killing. Figure S4. Prevalence of mutations in genetic pathways with immunomodulatory effects. (A) Prevalence of mutations in the key pathway for regulators of MHC-I regulation. (B) Prevalence of mutations in the key pathway for regulators for the response of cancer cells to NK-cell-mediated killing. (C) Prevalence of mutations in the key pathway for regulators for the response of cancer cells to macrophage-mediated killing. (D) Prevalence of mutations in the key pathway for regulators for the response of cancer cells to γδT-cell-mediated killing. Top panels show the prevalence of mutations in the key pathways for T cell killing to tumor cells in all PCAWG cancer samples. Bottom panels show the prevalence of mutations in the key pathways for T cell killing to tumor cells in different cancer types. Figure S5. Positive selection of mutations in genetic pathways [file media-1.zip › SFigures/SFigure6.pdf]

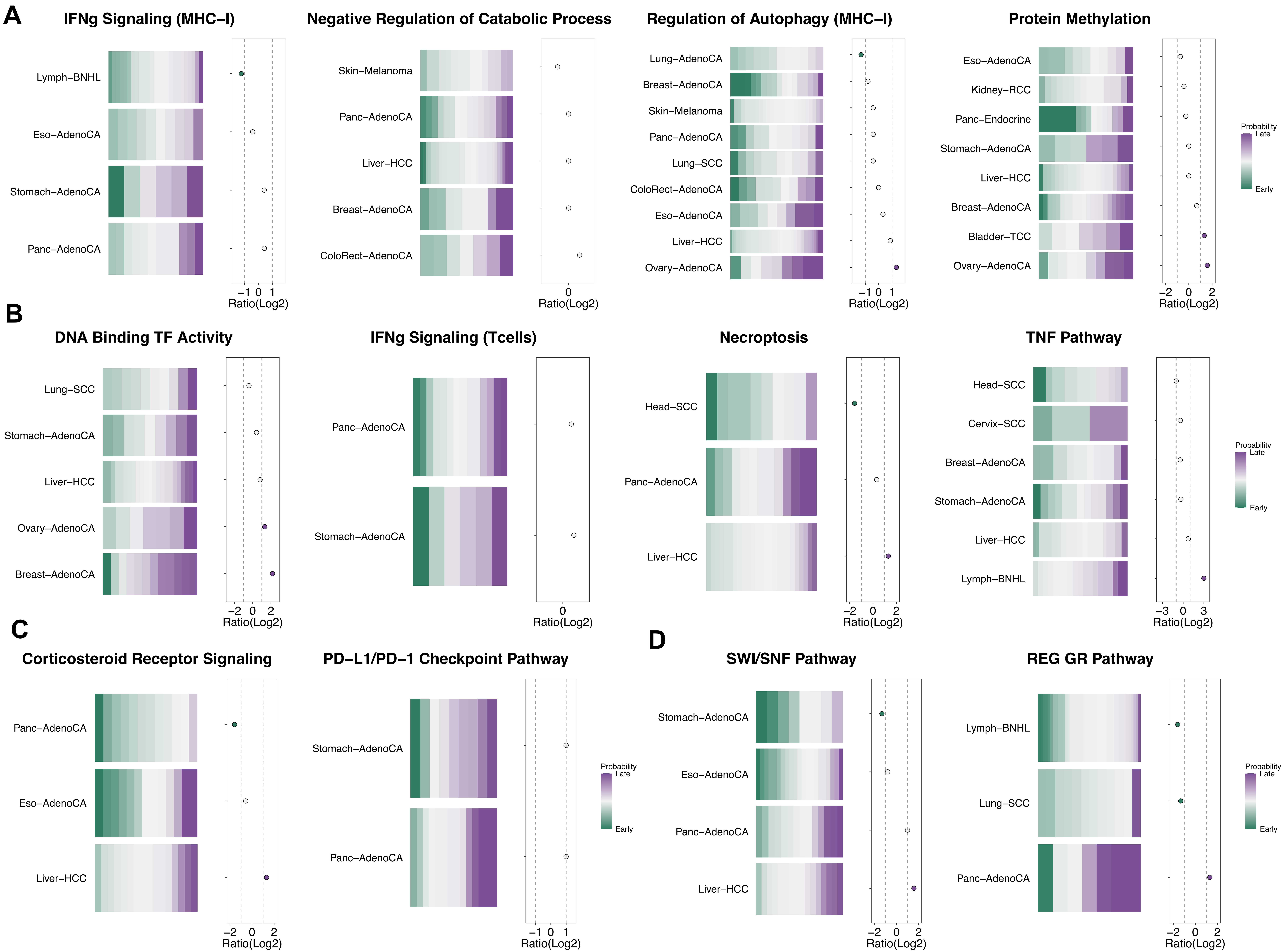
A
IFNg Signaling (MHC−I)
Lymph−BNHL
Eso−AdenoCA
Stomach−AdenoCA
Panc−AdenoCA
−1 0 1
Ratio(Log2)
Negative Regulation of Catabolic Process
Skin−Melanoma
Panc−AdenoCA
Liver−HCC
Breast−AdenoCA
ColoRect−AdenoCA
0
Ratio(Log2)
Regulation of Autophagy (MHC−I)
Lung−AdenoCA
Breast−AdenoCA
Skin−Melanoma
Panc−AdenoCA
Lung−SCC
ColoRect−AdenoCA
Eso−AdenoCA
Liver−HCC
Ovary−AdenoCA
−1 0 1
Ratio(Log2)
Protein Methylation
Eso−AdenoCA
Kidney−RCC
Panc−Endocrine
Stomach−AdenoCA
Liver−HCC
Breast−AdenoCA
Bladder−TCC
Ovary−AdenoCA
−2 0 2
Ratio(Log2)
Probability
Late
Early
B
DNA Binding TF Activity
Lung−SCC
Stomach−AdenoCA
Liver−HCC
Ovary−AdenoCA
Breast−AdenoCA
−2 0 2
Ratio(Log2)
IFNg Signaling (Tcells)
Panc−AdenoCA
Stomach−AdenoCA
0
Ratio(Log2)
Necroptosis
Head−SCC
Panc−AdenoCA
Liver−HCC
−2 0 2
Ratio(Log2)
TNF Pathway
Head−SCC
Cervix−SCC
Breast−AdenoCA
Stomach−AdenoCA
Liver−HCC
Lymph−BNHL
−3 0 3
Ratio(Log2)
Probability
Late
Early
C
Corticosteroid Receptor Signaling
Panc−AdenoCA
Eso−AdenoCA
Liver−HCC
−2 0 2
Ratio(Log2)
PD−L1/PD−1 Checkpoint Pathway
Stomach−AdenoCA
Panc−AdenoCA
−1 0 1
Ratio(Log2)
Probability
Late
Early
D
SWI/SNF Pathway
Stomach−AdenoCA
Eso−AdenoCA
Panc−AdenoCA
Liver−HCC
−2 0 2
Ratio(Log2)
REG GR Pathway
Lymph−BNHL
Lung−SCC
Panc−AdenoCA
−2 0 2
Ratio(Log2)
Probability
Late
Early
