# Supplementary material for: Evolutionary trajectories of immune escape across cancers
Source: bioRxiv. 2025 Jan 18:2025.01.17.632799. Preprint. [Version 1] doi: 10.1101/2025.01.17.632799 (PMC11761017; doi:10.1101/2025.01.17.632799)
Supplement: Supplement 1 — Figure S1. Overview of antigen presentation machinery mutations in PCAWG. (A) Total number of WGS samples included in the PCAWG dataset. (B) HLA mutation calling by Polysolver and HLA LOH calling by LOHHLA in PCAWG-TCGA samples. (C) dN/dS ratios for antigen presentation machinery mutations in each PCAWG cancer type. Figure S2. Temporal order of antigen presentation machinery mutations and key events in cancers. (A) Distribution of timing of antigen presentation machinery point mutation across cancer types. The plots show the mean timing of 250 timing samples for all APM mutations in each sample. (B) Distribution of timing of HLA LOH, and WGD across cancer types. Figure S3. Determining genetic pathways with immunomodulatory effects. (A) Summary of CRISPR screen studies reporting the enrichments of regulators for the response of cancer cells to NK-cell killing. (B) Summary of CRISPR screen studies reporting the enrichments of regulators for the response of cancer cells to macrophage killing. (C) Summary of CRISPR screen studies reporting the enrichments of regulators for the response of cancer cells to γδ T-cell killing. Figure S4. Prevalence of mutations in genetic pathways with immunomodulatory effects. (A) Prevalence of mutations in the key pathway for regulators of MHC-I regulation. (B) Prevalence of mutations in the key pathway for regulators for the response of cancer cells to NK-cell-mediated killing. (C) Prevalence of mutations in the key pathway for regulators for the response of cancer cells to macrophage-mediated killing. (D) Prevalence of mutations in the key pathway for regulators for the response of cancer cells to γδT-cell-mediated killing. Top panels show the prevalence of mutations in the key pathways for T cell killing to tumor cells in all PCAWG cancer samples. Bottom panels show the prevalence of mutations in the key pathways for T cell killing to tumor cells in different cancer types. Figure S5. Positive selection of mutations in genetic pathways [file media-1.zip › SFigures/SFigure20.pdf]

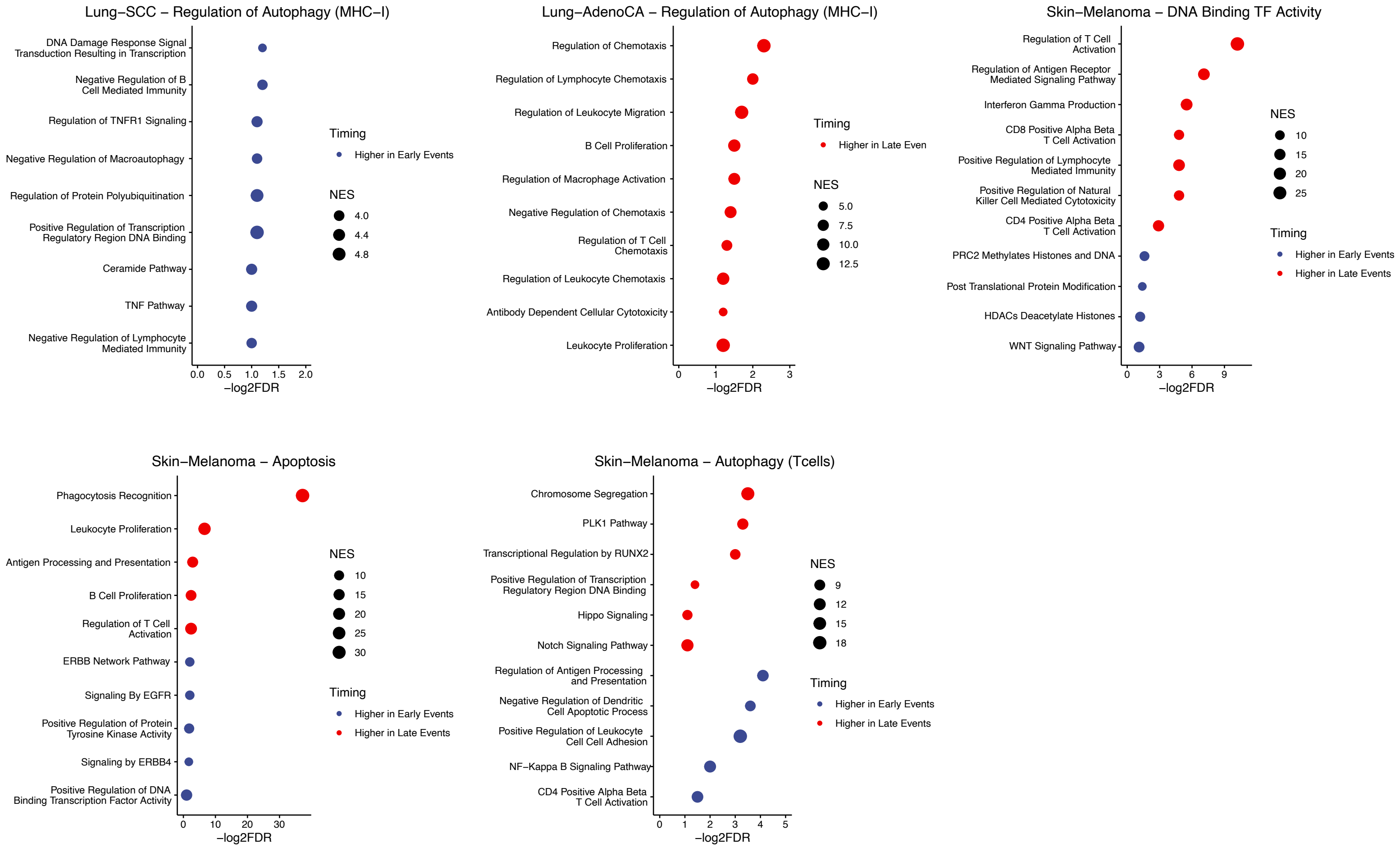

Lung–SCC – Regulation of Autophagy (MHC–I)
DNA Damage Response Signal Transduction Resulting in Transcription
Negative Regulation of B Cell Mediated Immunity
Regulation of TNFR1 Signaling
Negative Regulation of Macroautophagy
Regulation of Protein Polyubiquitination
Positive Regulation of Transcription Regulatory Region DNA Binding
Ceramide Pathway
TNF Pathway
Negative Regulation of Lymphocyte Mediated Immunity
0.0 0.5 1.0 1.5 2.0
–log2FDR
Timing
Higher in Early Events
NES
4.0
4.4
4.8
Lung–AdenoCA – Regulation of Autophagy (MHC–I)
Regulation of Chemotaxis
Regulation of Lymphocyte Chemotaxis
Regulation of Leukocyte Migration
B Cell Proliferation
Regulation of Macrophage Activation
Negative Regulation of Chemotaxis
Regulation of T Cell Chemotaxis
Regulation of Leukocyte Chemotaxis
Antibody Dependent Cellular Cytotoxicity
Leukocyte Proliferation
0 1 2 3
–log2FDR
Timing
Higher in Late Even
NES
5.0
7.5
10.0
12.5
Skin–Melanoma – DNA Binding TF Activity
Regulation of T Cell Activation
Regulation of Antigen Receptor Mediated Signaling Pathway
Interferon Gamma Production
CD8 Positive Alpha Beta T Cell Activation
Positive Regulation of Lymphocyte Mediated Immunity
Positive Regulation of Natural Killer Cell Mediated Cytotoxicity
CD4 Positive Alpha Beta T Cell Activation
PRC2 Methylates Histones and DNA
Post Translational Protein Modification
HDACs Deacetylate Histones
WNT Signaling Pathway
0 3 6 9
–log2FDR
NES
10
15
20
25
Timing
Higher in Early Events
Higher in Late Events
Skin–Melanoma – Apoptosis
Phagocytosis Recognition
Leukocyte Proliferation
Antigen Processing and Presentation
B Cell Proliferation
Regulation of T Cell Activation
ERBB Network Pathway
Signaling By EGFR
Positive Regulation of Protein Tyrosine Kinase Activity
Signaling by ERBB4
Positive Regulation of DNA Binding Transcription Factor Activity
0 10 20 30
–log2FDR
NES
10
15
20
25
30
Timing
Higher in Early Events
Higher in Late Events
Skin–Melanoma – Autophagy (Tcells)
Chromosome Segregation
PLK1 Pathway
Transcriptional Regulation by RUNX2
Positive Regulation of Transcription Regulatory Region DNA Binding
Hippo Signaling
Notch Signaling Pathway
Regulation of Antigen Processing and Presentation
Negative Regulation of Dendritic Cell Apoptotic Process
Positive Regulation of Leukocyte Cell Cell Adhesion
NF–Kappa B Signaling Pathway
CD4 Positive Alpha Beta T Cell Activation
0 1 2 3 4 5
–log2FDR
NES
9
12
15
18
Timing
Higher in Early Events
Higher in Late Events
